# Improving Adherence to Essential Birth Practices Using the WHO Safe Childbirth Checklist With Peer Coaching: Experience From 60 Public Health Facilities in Uttar Pradesh, India

**DOI:** 10.9745/GHSP-D-16-00410

**Published:** 2017-06-27

**Authors:** Megan Marx Delaney, Pinki Maji, Tapan Kalita, Nabihah Kara, Darpan Rana, Krishan Kumar, Jenny Masoinneuve, Simon Cousens, Atul A Gawande, Vishwajeet Kumar, Bhala Kodkany, Narender Sharma, Rajiv Saurastri, Vinay Pratap Singh, Lisa R Hirschhorn, Katherine EA Semrau, Rebecca Firestone

**Affiliations:** a Ariadne Labs, a Joint Center between Brigham and Women's Hospital and the Harvard T.H. Chan School of Public Health, Boston, MA, USA.; b Population Services International, Lucknow, Uttar Pradesh, India.; c London School of Hygiene & Tropical Medicine, London, UK.; d Department of Health Policy and Management, Harvard T.H. Chan School of Public Health, Boston, MA, USA.; e Department of Surgery, Brigham and Women's Hospital, Boston, MA, USA.; f Community Empowerment Lab, Lucknow, Uttar Pradesh, India.; g Jawaharlal Nehru Medical College, Karnataka, India.; h Feinberg School of Medicine, Northwestern University, Chicago, IL, USA.; i Department of Medicine, Harvard Medical School, Boston, MA, USA.; j Division of Global Health Equity, Department of Medicine, Brigham and Women's Hospital, Boston, MA, USA.; k Population Services International, Washington, DC, USA.

## Abstract

**Background:**

Adherence to evidence-based essential birth practices is critical for improving health outcomes for mothers and newborns. The WHO Safe Childbirth Checklist (SCC) incorporates these practices, which occur during 4 critical pause points: on admission, before pushing (or cesarean delivery), soon after birth, and before discharge. A peer-coaching strategy to support consistent use of the SCC may be an effective approach to increase birth attendants' adherence to these practices.

**Methods:**

We assessed data from 60 public health facilities in Uttar Pradesh, India, that received an 8-month staggered coaching intervention from December 2014 to September 2016 as part of the BetterBirth Trial, which is studying effectiveness of an SCC-centered intervention on maternal and neonatal harm. Nurse coaches recorded birth attendants' adherence to 39 essential birth practices. Practice adherence was calculated for each intervention month. After 2 months of coaching, a subsample of 15 facilities was selected for independent observation when the coach was not present. We compared adherence to the 18 practices recorded by both coaches and independent observers.

**Results:**

Coaches observed birth attendants' behavior during 5,971 deliveries. By the final month of the intervention, 35 of 39 essential birth practices had achieved >90% adherence in the presence of a coach, compared with only 7 of 39 practices during the first month. Key behaviors with the greatest improvement included explanation of danger signs, temperature measurement, assessment of fetal heart sounds, initiation of skin-to-skin contact, and breastfeeding. Without a coach present, birth attendants' average adherence to practices and checklist use was 24 percentage points lower than when a coach was present (range: −1% to 62%).

**Conclusion:**

Implementation of the WHO Safe Childbirth Checklist with coaching improved uptake of and adherence to essential birth practices. Coordination and communication among facility staff, as well as behaviors with an immediate, tangible benefit, showed the greatest improvement. Difficult-to-perform behaviors and those with delayed or theoretical benefits were less likely to be sustained without a coach present. Coaching may be an important component in implementing the Safe Childbirth Checklist at scale. (After publication of this article, the impact results of the BetterBirth intervention were published in the New England Journal of Medicine [volume 377, pages 2313-2324, doi: 10.1056/NEJMoa1701075]. The results showed that the intervention had no significant effect on maternal or perinatal mortality or maternal morbidity, despite having positive effects on essential birth practices.)

## Abstract (precis)

Implementation of the WHO Safe Childbirth Checklist with peer coaching resulted in >90% adherence to 35 of 39 essential birth practices among birth attendants after 8 months, but adherence to some practices was lower when the coach was absent.

See related article by Kara.

## BACKGROUND

Childbirth and the first 24 hours postpartum remains a precarious time for both mother and newborn despite improvements in maternal and neonatal mortality over the past 2 decades.^1^^–^^3^ Previously, poor outcomes were thought to result primarily from childbirth occurring outside of health care facilities and from lack of access to skilled care. However, strategies such as the Janani Suraksha Yojana (JSY) program in India have increased rates of facility-based childbirth without significantly decreasing maternal and neonatal mortality.^4^^–^^7^ Thus, improving the quality of care provided during facility-based childbirth is a key strategy to decrease maternal and neonatal mortality globally.^8^ The World Health Organization (WHO) *Quality of Care Framework for Maternal and Neonatal Health* describes quality care as being safe, effective, timely, efficient, equitable, and people-centered.^9^ Use of evidenced-based practices for routine care and management of complications is key to achievement of high quality of care.^10^

Essential birth practices that reduce harm and save lives during childbirth are well documented, but all too often, they are not performed. Other areas of health care have used a checklist-based approach to address this “know-do” gap.^11^^,^^12^ WHO's Safe Childbirth Checklist (SCC), developed in 2009, is a low-cost tool that codifies these essential birth practices in a format designed to be accessible to birth attendants to ensure that timely, lifesaving practices are performed for every facility-based birth, thus improving the quality of care.^13^^,^^14^

Essential birth practices that reduce harm and save lives during childbirth are well documented but often not performed.

Checklists are job aids designed to support routine adherence to evidence-based practices, and as such are intended to change health care workers' behaviors.^15^ Job aids alone have not been found to improve health care workers' performance,^16^ and experience with implementing checklists has demonstrated that additional strategies are needed to promote behavior change.^17^ Previous studies of the SCC have suggested that peer coaching based on feedback about SCC use,^18^ ensuring buy-in from the larger health care system, and integrating the SCC into existing workflows^19^ increased the likelihood of making real, sustained improvements in the quality of facility-based childbirth care. The BetterBirth Program—an intervention aiming for sustained SCC adoption through coaching-based implementation—was developed based on lessons learned from early implementations of the SCC and from other quality-improvement projects.^20^^,^^21^

We aimed to assess whether the behavior change intervention of this SCC-based peer-coaching program was associated with improved performance of essential birth practices during facility-based childbirth care. First, we examined birth attendants' adherence to a set of essential birth practices in 60 intervention facilities, as observed by a coach. Then, in a subset of these facilities, we employed independent observers to verify adherence to these practices in the absence of the coach.

We aimed to assess whether implementation of the WHO Safe Childbirth Checklist with peer coaching was associated with improved performance of essential birth practices.

## METHODS

### Study Design

The BetterBirth Trial was a matched-pair, cluster-randomized controlled trial conducted in Uttar Pradesh, India, to test whether the introduction of the SCC paired with peer coaching—the BetterBirth Program—could reduce maternal morbidity and mortality and perinatal mortality in 120 health care facilities (60 intervention facilities and 60 control facilities receiving standard of care) (ClinicalTrials.gov: NCT2148952; Universal Trial Number: U1111-1131-5647). The methodology of the trial is available elsewhere,^20^ and a detailed description of the intervention is published as a companion article in this issue of *Global Health: Science and Practice*.^21^ Impact results on perinatal mortality, maternal mortality, and severe maternal morbidity will be reported elsewhere.

### Intervention: The BetterBirth Program

The BetterBirth Program supported adoption and use of the SCC using a 3-pronged implementation pathway (Box)^21^:

1. Engagement with facility leaders and government officials at the district and state level
2. A 2-day motivational launch event of the SCC for facility staff
3. Support to birth attendants, facility leaders, and government officials through visitation of peer coaches to give regular feedback

The BetterBirth Program used a 3-pronged approach to support adoption and use of the WHO Safe Childbirth Checklist: engagement with key stakeholders, a motivational launch event, and peer-coaching support to birth attendants.

BOX **The BetterBirth Intervention**

**TOOLS**

**The World Health Organization Safe Childbirth Checklist (SCC)** comprises 28 essential birth practices to improve the quality of labor and delivery care.

**Pulse** is an electronic data management system for quality improvement. Coaches entered their observations of birth attendants' behavior into Pulse via mobile-phone apps; Pulse then generated real-time “heat maps” to guide facility staff to identify gaps in care and find effective solutions.

**STRATEGY: COACHING FOR EMPOWERMENT**

Goals of coaching:

- Motivating birth attendants to change their practices.
- Observing, recording, and feeding back information about birth attendants' behavior.
- Supporting birth attendants to problem solve and resolve barriers to essential practices.

Principles of coaching:

- Multilevel: Coaches worked with birth attendants, and coach team leaders worked with facility and district leadership to problem solve and facilitate change across the health system.
- Collaborative: Coaches and birth attendants had supportive, constructive, respectful, peer-to-peer relationships.
- Provider-centered: Coaches responded to the needs of the birth attendants and facility and district leaders with whom they worked rather than following a predetermined agenda.

**IMPLEMENTATION: ENGAGE-LAUNCH-SUPPORT**

- **Engage:** Program goals and strategies were introduced to leadership at the national, state, and district level.
- **Launch:** A motivational event at each facility introduced the significance of the tools, explained the coaching strategy, and enlisted the participation of staff in a needs assessment.
- **Support:** Nurse coaches supported SCC adoption and quality improvement by making 43 visits to each facility over 8 months (twice weekly for months 1–4; once weekly for months 5–6; fortnightly for month 7; and monthly for month 8). Coach team leaders, who were physicians or public health professionals, accompanied coaches on half of the visits in order to address system-level and supply issues at the facility.

**SUSTAINABILITY PLAN**

A motivated and respected staff member was selected by facility leadership to be a childbirth quality coordinator (CQC) at each facility. The CQC worked closely with team leaders to improve quality of care and champion the SCC beyond the BetterBirth Program.

For a detailed description of the intervention, see Kara, Firestone, Kalita, et al. (2017).^21^

The intervention used a coaching strategy as the primary mechanism to encourage behavior change among health care workers. Coaches were trained nurses who worked directly with birth attendants. Coach team leaders were trained physicians or public health leaders who worked directly with facility leaders and government officials and who supervised the nurse coaches. Coaches visited the facility twice weekly during the early stages of the intervention, with the frequency of visits decreasing to once monthly by the end of the intervention, for a total of 43 visits over an 8-month period. Coaches did not provide patient care. Although coaches did not clinically intervene in emergencies, if needed they could escalate emergency situations to be addressed by a medical officer at the facility. Additionally, coaches had the discretion to encourage appropriate referral of a mother or a baby before, during, or after patient observation, as this is directly related to the SCC.

Birth attendants were coached to use the SCC at 4 critical “pause points” during childbirth:

1. On admission
2. Before pushing or before cesarean delivery
3. Soon after birth (within 1 hour)
4. Before discharge

The paper-based checklist itself was commonly attached to a mother's chart or bedhead ticket for ease of reference and so completed tasks could be checked off for each patient at each pause point. Posters of the SCC were also posted on the wall in the delivery area.

The BetterBirth intervention used a behavior change framework to facilitate the adherence to checklist-based essential practices.^22^^,^^23^ The core of this framework is characterized by coaching birth attendants and leaders to recognize gaps in essential birth practices, barriers to delivering them, and solutions for overcoming those barriers. To support ongoing facility-level adherence to essential practices, a facility staff member was identified and trained to support SCC use after the intervention was completed. The program is described in more detail elsewhere.^18^^,^^20^^,^^21^ Technical training and supplies were not provided. Instead, coaches and team leaders worked with facility staff to resolve these barriers through existing channels. Control sites in the BetterBirth Trial received the standard of care. Although Indian national guidelines recommend use of the SCC,^24^ we did not observe its use at any control facilities throughout the trial.

### Setting and Site Selection

Uttar Pradesh, India's most populous state, has some of the highest maternal mortality ratios and neonatal mortality rates.^25^ Across the state, 773 community health centers (CHCs) and 3,497 primary health centers (PHCs) serve as public-sector facilities to provide health services, including obstetric care, for nearly 200 million inhabitants of the state.^25^ Patients with major complications needing a cesarean delivery or blood transfusion are referred to a district hospital or a CHC first referral unit (CHC-FRU).

The BetterBirth Program was implemented in 60 public-sector facilities, including PHCs, CHCs, and CHC-FRUs, across 24 districts of Uttar Pradesh from December 2014 to September 2016. Site selection for the overall trial and batch-wise implementation is described elsewhere.^20^ Of the 60 intervention facilities, a pragmatic sample of 15 facilities was selected based on geographic location, in which independent observers recorded birth attendant behavior when coaches were absent.

### Outcomes of Interest

For the primary analysis, we operationalized the 28 items on the SCC into 43 discrete measures (39 essential practices related to patient care and supply preparation plus 4 measures of checklist use) (Table 1), as some of the items on the checklist require multiple steps. For example, the first checklist item—”Does mother need referral?”—requires a birth attendant to separately measure temperature to assess for fever, to measure blood pressure to assess for preeclampsia, and to assess the fetal heart sounds to detect fetal distress. We selected measures that could be clearly observed by a coach who was standing nearby.

**TABLE 1.** Essential Birth Practices Observed by Peer Coaches, by SCC Pause Point^a^

Pause Point 1: On Admission	Pause Point 2: Before Pushing	Pause Point 3: Within 1 Hour of Delivery	Pause Point 4: Before Discharge
**Mother's temperature on admission** **Mother's blood pressure on admission** Measurement of fetal heart sounds Vaginal exam done If yes, hand hygiene before exam (soap and water or alcohol rub) If yes, gloves worn for exam Danger signs explained to mother or birth companion at admission **Checklist use at admission**	Mother's temperature before delivery Mother's blood pressure before delivery **Clean towel available at bedside** Gloves available at bedside **Pads available at bedside** Oxytocin available at bedside **Blade available at bedside** **Cord ligature available at bedside** **Mucus extractor available at bedside** **Neonatal bag and mask available at bedside** **Hand hygiene before delivery (soap and water or alcohol rub)** **Checklist use before delivery**	**Glove use at birth** Was baby breathing assessed at birth? **Skin-to-skin immediately after birth** **Oxytocin given 1 minute after birth** Check bleeding after delivery Mother's temperature after delivery Mother's blood pressure after delivery **Baby's temperature after delivery** **Baby's weight** **Breastfeeding initiation** Danger signs explained to mother or birth companion after delivery Skin-to-skin at 1 hour **Checklist use after delivery**	Check bleeding before discharge Mother's temperature before discharge Baby's temperature before discharge Check baby breathing before discharge Check baby feeding before discharge BCG vaccine given OPV given Family planning discussed Danger signs explained to mother or birth companion before discharge Checklist use before discharge

Abbreviations: BCG, bacille Calmette-Guérin; OPV, oral polio vaccine; SCC, Safe Childbirth Checklist.

a Bolded practices (n=18) were observed by both coaches and independent observers.

For the subanalysis of 15 facilities conducted after the first 2 months of coaching were completed, the outcome of interest was the difference between birth attendants' adherence to essential birth practices in the presence of a coach and birth attendants' behavior documented by independent observers in the absence of the coach. Since not all observed behaviors were recorded in the same way by the coaches and independent observers due to differences in data collection procedures, we included only the 18 overlapping checklist-related behaviors in the analysis (Table 1).

### Data Collection

#### Coach Observation

Coaches used standardized tools to document SCC use and adherence to essential birth practices at the 4 pause points listed on the SCC. Deliveries were selected for observation based on if a patient was present and a birth attendant was available to be observed for at least 1 complete pause point. Birth attendants could be observed for 1 or more pause points at each delivery. During the 8-month intervention, coaches attempted to observe each birth attendant at a facility multiple times. Data were first collected on paper forms while observing childbirth and subsequently entered into a mobile phone-based CommCare app (Dimagi, Cambridge, MA) on the same day but after the coach left the patient care area. Practices were coded as either “completed” (green), “completed after prompt” (yellow), or “not completed” (red) and transformed into a standardized heat map report, displayable on a mobile device for coaches and team leaders to report back to birth attendants and facility leadership on adherence to the SCC and to essential birth practices.^21^

#### Independent Observation

For the subanalysis, independent observers assessed SCC use and birth attendants' adherence to essential birth practices. Starting during the eighth week of coaching, independent observers visited facilities on non-coaching days to record adherence to essential practices. Independent observers collected data for a period of 6 to 12 weeks, depending on the delivery load of the facility, with a goal to reach 240 pause point observations per facility. Independent observers selected any case for which a pause point could be observed from start to finish. A mother was observed for as many pause points as possible. Data collected by independent observers were considered a proxy for birth attendant behavior under everyday conditions at the facility.

Independent observers were nurses trained in childbirth who used a standardized tool to record behavioral data. Intensive training to ensure data quality was provided.^26^ Similar to coaches, independent observers recorded data on all behaviors within a specific pause point; birth attendants could be observed for 1 or more pause points. Due to differences in how facilities handled the process of patient discharge, independent observers did not observe the fourth pause point of the SCC (“before discharge”). Independent observers also first recorded data on paper forms and subsequently entered the data into an app on the same day after leaving the patient care area. Data collected by independent observers were not shared with facility staff. Moreover, in emergency situations, independent observers did not provide care nor did they intervene to facilitate a response.

### Data Analysis

We analyzed adherence to each of the 43 coach-observed practices by month over the 8-month intervention. First, we calculated an adherence proportion for each behavior based on the number of times a behavior was completed divided by the number of times that a behavior was expected to be completed at a given pause point. We then plotted this proportion across the 8 months of the intervention to understand how adherence to essential practices changed over time.

Since coaches visited facilities only fortnightly or monthly by the seventh and eighth months of the intervention, data from these 2 months were combined (represented as Month 7). We used percentage-point differences to compare adherence during Month 7 versus Month 1 and allocated behaviors into 3 categories:

1. Minimal improvement (<15 percentage-point absolute difference)
2. Moderate improvement (15 to 24 percentage-point absolute difference)
3. Major improvement (≥25 percentage-point absolute difference)

We developed a logistic regression model for each behavior to assess the probability of birth attendants' adherence to a given behavior across the 8 months of the intervention, controlling for facility clustering using dummy variables for facility. We calculated odds ratios and 95% confidence intervals to test how adherence changed over time. Results were considered statistically significant at *P*<.05.

For the subanalysis, we calculated adherence to each of the 18 measured practices recorded by both nurse coaches and independent observers. We used percentage-point differences to compare adherence when a coach was present versus absent. Each behavior was categorized as having a:

1. Minimal difference (<15 percentage-point absolute difference)
2. Moderate difference (15 to 24 percentage-point absolute difference)
3. Major difference (≥25 percentage-point absolute difference)

A Rao-Scott chi-square test was used to adjust for clustering within facility when comparing the overall proportion of practices completed between coaches and independent observers. We also compared adherence rates reported by coaches at the 15 sites of the subanalysis with the 45 sites not included in the subanalysis to assess differences between the 2 groups. All data analyses were conducted using Stata SE 13.1 for Mac and Microsoft Excel 2011.

### Qualitative Study to Explore Explanatory Factors

As a strategy to improve the quality and effectiveness of the BetterBirth Program, we used an explanatory qualitative study. We presented adherence rates documented by coaches and independent observers to members of the field implementation staff during a week-long workshop in September 2016. Successes and challenges related to birth attendants' adherence to individual behaviors were discussed and documented.

### Ethics Approval and Consent Process

The BetterBirth Trial, including data for this analysis, was approved by the Harvard T.H. Chan School of Public Health Institutional Review Board, the World Health Organization Ethics Review Board, the Population Services International Research Ethics Board, the Community Empowerment Labs Ethics Review Committee, and the Jawaharlal Nehru Medical College-Belgaum Ethics Review Board. All activities were conducted in partnership with the Government of Uttar Pradesh.

For coaching, each facility and birth attendant formally agreed to participate in the BetterBirth Program as a quality improvement initiative at the beginning of the intervention. Coaches accompanied birth attendants during their work shift and documented practices during patient-care activities at the facility. Coaches did not collect any patient identifiers.

For independent observation, each facility and birth attendant formally agreed to participate in the study. Since these data were collected solely for the purpose of research as part of the larger BetterBirth Trial, and identifying information of the mother was collected, a more rigorous consent process was used. Prior to each observation, independent observers verbally confirmed that the birth attendant agreed to be observed. Patients signed written consent to have independent observers present during their care.

## RESULTS

### Coach Observers

**FIGURE 1:**
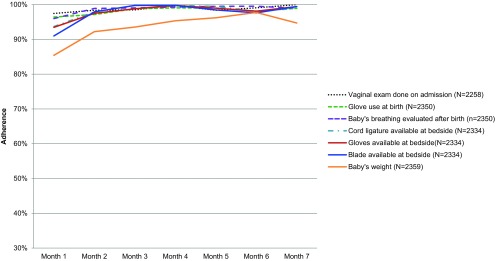
Essential Birth Practices Consistently Performed by Birth Attendants in 60 Facilities Across the 8-Month BetterBirth Intervention, Uttar Pradesh, India

In 60 public health facilities during 8 months of intervention, coaches observed care provided by birth attendants during 5,971 deliveries at 1 or more pause points during childbirth. Additional facility characteristics and intervention process measures are available in Supplement 1. By the final month of the intervention, 35 of 39 essential practices had achieved >90% adherence in the presence of a coach (Supplement 2), compared with only 7 of 39 practices that had achieved this level of adherence during the first month (Figure 1). Throughout the intervention, coaches observed consistently high adherence to the preparation of birth supplies at the bedside, with nearly 100% adherence noted by the second month of coaching.

35 of 39 essential birth practices had achieved >90% adherence in the presence of a coach by the final month of the intervention.

**FIGURE 2:**
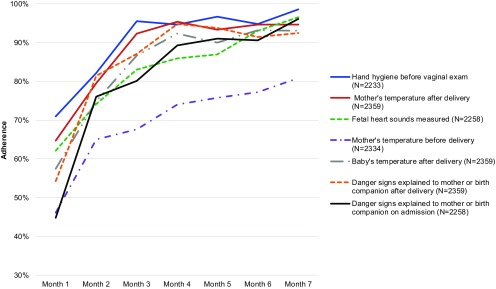
Essential Birth Practices With the Greatest Increase in Adherence by Birth Attendants in 60 Facilities Across the 8-Month BetterBirth Intervention, Uttar Pradesh, India

Essential birth practices with the greatest absolute increase in adherence over time included explaining danger signs to the mother or to her birth companion on admission (45% to 96%; *P*<.001) and after delivery (54% to 92%; *P*<.001), measurement of baby's temperature (57% to 93%; *P*<.001), measurement of mother's temperature before (46% to 81%; *P*<.001) and after delivery (65% to 95%; *P*<.001), and measurement of fetal heart sounds on admission (62% to 97%; *P*<.001) (Figure 2 and Supplement 2). More moderate increases were seen across other practices, including oxytocin administration within 1 minute of delivery (81% to 99%; *P*<.001), skin-to-skin infant care (71% to 94%; *P*<.001), and hand hygiene before delivery (76% to 94%; *P*<.001). Across all 39 behaviors, improvements ranged from an absolute increase of 2 percentage points to 51 percentage points from the first to the final month.

Behaviors that did not attain an adherence rate of 90% or above for any month of the intervention included measurement of a mother's temperature (46% to 81%; *P*<.001) and blood pressure (53% to 80%; *P*<.001) before delivery, as well as administration of bacille Calmette-Guérin (BCG) vaccine (75% to 87%; *P*<.001) and the oral polio vaccine (OPV) (86% to 89%; *P*<.001) to an infant before discharge.

Checklist use at each pause point increased between the first and final month of coaching, including checklist use on admission (84% to 98%; *P*=.002), before delivery (66% to 94%; *P*<.001), after delivery (75% to 95%; *P*<.001), and before discharge (90% to 99%; *P*<.001).

Behaviors that did not reach >90% adherence consisted of measurement of a mother's temperature and blood pressure before delivery and administration of BCG and oral polio vaccines to an infant before discharge.

### Independent Observers

**FIGURE 3:**
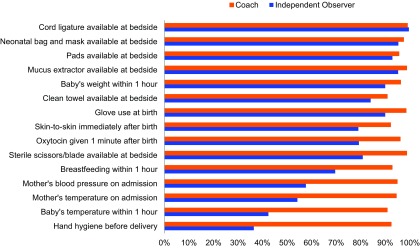
Adherence to Essential Birth Practices by Birth Attendants in 15 Facilities, as Observed by Coaches Versus Independent Observers (With Coaches Absent) After 2 Months of the BetterBirth Intervention, Uttar Pradesh, India

In the subset of 15 facilities, independent observers documented essential practices after 2 months of coaching on 1,277 deliveries at 1 or more pause points, while coaches observed 736 deliveries over the same 12-week period in the same facility. There was an absolute difference of 24 percentage points (range: −1 to 62 percentage points) in the proportion of the 18 practices completed when the coach was present versus absent (Figure 3 and Supplement 3). Of the essential birth practices recorded by both coaches and independent observers, a minimal absolute difference (<15 percentage points) in levels of adherence was observed for preparation of supplies including cord ligature, neonatal bag and mask, mucus extractor, pads and clean towel, weighing of the baby, glove use during delivery, and immediate skin-to-skin care. A moderate absolute difference (15 to 24 percentage points) was observed across 3 behaviors, including practices such as oxytocin administration within 1 minute of birth and breastfeeding within 1 hour of birth. A major absolute difference (>25 percentage points) was seen with hand hygiene, measuring the baby's temperature, and measuring the mother's blood pressure and temperature. We found no major differences between the coach-recorded adherence rate in the 15 facilities of the subanalysis versus the 45 facilities not in the subanalysis.

There was an absolute difference of 24 percentage points in the proportion of the 18 practices completed when a coach was present versus absent.

Through the explanatory exercise conducted with the BetterBirth Program implementation team, we documented experiences from the field related to coaching on various behaviors (Table 2). In particular, many of the practices with the greatest improvements in adherence were those in which the birth attendants saw tangible benefits to implementing them, such as checking the mother for bleeding after delivery to recognize hemorrhage early, when it is easier to treat, and initiating immediate skin-to-skin contact between baby and mother to better regulate temperature and more easily initiate breastfeeding. Those practices with minimal improvement, namely, BCG vaccine and OPV administration, may have been due to incentives to minimize waste; for example, each BCG vial contained 10 doses, so coaches observed birth attendants avoiding administration unless a certain number of babies was present to avoid wasting vaccines.

**TABLE 2.** Implementation Experience of Coaches and Independent Observers on Implementing the WHO Safe Childbirth Checklist in Uttar Pradesh, India

Summary of Coach-Observed Adherence Over Intervention Period	Essential Birth Practice Example	Average Adherence Level Observed by Coach	Absolute Difference Over Time (Observed by Coach)	Qualitative Summary of Coaches' Implementation Experience	Summary of Independent Observers' Findings
Minimal improvement (<15 percentage points) due to high initial adherence	Supply preparation before delivery (gloves, cord ligature, blade)	98%, 98%, 97%	6%, 5%, 8%	Coaches used the SCC to encourage birth attendants and labor room staff to prepare and organize materials prior to deliveries or early in the day so that supplies were ready to use.	Supply preparation remained consistent even when the coach was absent
	Measuring baby's weight after birth	93%	9%	Measuring a baby's weight is a standard requirement in birth registries and used to calculate Vitamin K dosage, thus weight was frequently taken. Additional pressure from families to know a baby's birth weight contributed to high adherence.	Measuring a baby's weight remained consistent even when the coach was absent.
Minimal improvement (<15 percentage points) achieved	BCG vaccine administration	77%	13%	Incentives at the facility and district level to minimize waste may have contributed to less consistent administration of BCG and other vaccines. Each BCG vial contained 10 doses; birth attendants were observed to avoid administration unless a certain number babies were present to avoid wasting vaccines.	N/A (not measured).
	Oral polio vaccine administration	87%	3%		
Moderate improvement (15 to 24 percentage-point absolute difference)	Hand hygiene before delivery	90%	18%	Coaches found that hand hygiene was more consistently done before delivery, compared with before a vaginal exam during admission.	This behavior saw the greatest difference between coach and independent observer (92% vs. 36%).
	Oxytocin administration within 1 minute of delivery	92%	19%	Birth attendants noticed the effects of changing the timing and route of oxytocin administration—from IV administration to augment labor to IM administration immediately postpartum—which they felt contributed to decreased hemorrhage and decreased fetal distress.	Moderate absolute difference (17 percentage points) when the coach was not present.
	Skin-to-skin immediately after birth	87%	23%	Coaches observed that birth attendants appreciated tangible improvements in babies' status from initiating skin-to-skin immediately, including better temperature regulation and easier initiation of breastfeeding.	Minimal absolute difference (13 percentage points) when the coach was not present.
Greatest improvement (≥25 percentage-point absolute difference)	Check mother for bleeding after delivery	89%	26%	Coaches noted that birth attendants saw the value of routinely assessing bleeding in order to recognize hemorrhage early, when it is easier to treat.	N/A (not measured).
	Initiation of breastfeeding	87%	27%	Coaches felt that they were able to reinforce the importance of this practice due to the clear governmental guidelines that promote breastfeeding.	Moderate absolute difference (23 percentage points) when the coach was not present.
	Skin-to-skin at 1 hour	83%	29%	If skin-to-skin was not initiated immediately, coaches found it difficult to gain commitment to this practice, as birth attendants faced competing priorities of needing to complete birth-related paperwork and families' pressure to show the newborn to relatives waiting outside of the labor room.	N/A (not measured).
	Temperature measurement after delivery (mother, baby)	86%, 81%	30%, 36%	Birth attendants commonly used their hand to subjectively feel if a patient had a fever and were satisfied with this method. Thermometers may have been broken or misplaced. Many facilities experienced unreliable electricity, and thermometers were difficult to read in dark rooms. Coaches found that it was challenging to gain commitment to this behavior.	Major absolute difference in measurement of baby's temperature (48 percentage points) when the coach was not present. Independent observers did not document mothers' temperature after delivery.
Variable improvement in checklist use	Checklist use On admission Before delivery After delivery Before discharge	94% 87% 92% 97%	14% 28% 21% 9%	More structured patient assessments that occurred on admission and within 1 hour after birth were conducive to SCC use. Just before delivery was an extremely busy time for birth attendants; birth attendants frequently regarded referring to a checklist as more of a burden or barrier to providing timely care at pause point 2. Because the SCC was a standalone document and not integrated into the existing patient record (bedhead ticket), it was easy to overlook. Coaches saw the importance of advocating to the heads of facilities to integrate the SCC into the bedhead ticket.	Moderate to major absolute difference when the coach was not present (38 percentage-point difference in checklist use on admission, 62 percentage-point difference before delivery, 21 percentage-point difference after delivery). Independent observers were not present at discharge.

Abbreviations: BCG, bacille Calmette-Guérin; IM, intramuscular; IV, intravenous; SCC, Safe Childbirth Checklist; WHO, World Health Organization.

## DISCUSSION

Implementation of the WHO Safe Childbirth Checklist with peer coaching and data feedback was associated with improved uptake of essential birth practices among birth attendants in the presence of a coach. We found greater than 90% adherence by the final month of the intervention for 35 of 39 essential birth practices when a coach was present, compared with only 7 of 39 practices during the first intervention month. However, when coaches were not present, independent observers noted an average absolute difference of 24 percentage points in adherence in a subset of behaviors after 2 months of coaching.

Pilot testing of SCC implementation using a multilevel coaching approach in Karnataka, India, which served as a model for the larger BetterBirth Program in Uttar Pradesh, found similar improvements in the overall number of essential birth practices completed.^27^ Other coaching or nurse mentoring programs designed to improve facility-based childbirth care have additionally included program-provided birth-related supplies^28^ or technical training^29^ while requiring relatively fewer coaching visits; these programs have found similar increases in the number of essential practices performed by birth attendants, although strategies to measure practice adherence varied across studies. Other attempts to improve the quality of childbirth using the SCC without peer coaching, such as in a tertiary hospital in Sri Lanka, found poor levels of adoption.^30^

Using a coaching-based implementation of the SCC in Uttar Pradesh, we have identified a number of themes that may account for the patterns of improvement observed, including degree of change and level of adoption, which may have broader implications for implementing the SCC in other settings.

### Behaviors With Highly Visible Benefits

Health care workers do not want to cause harm and are often reluctant to try new ways of doing things.^31^ Available evidence suggests that experiencing immediate, visible benefits from a new practice increases the likelihood that an individual will repeat the new practice; this visibility can support behavior change and habit formation.^32^^,^^33^ For birth attendants in intervention facilities, essential birth practices with tangible benefits were more easily incorporated into routine practice. These early wins helped to increase birth attendants' interest and commitment to incorporating essential practices on the SCC into their daily routines. For example, soon after initiation of coaching, birth attendants in many facilities reported switching their use of oxytocin from intravenous administration to augment labor to intramuscular administration immediately postpartum, which they felt reduced the incidence of hemorrhage and fetal distress. This represents a lifesaving improvement in care and complies with WHO guidelines for the Active Management of the Third Stage of Labor (AMTSL) that every woman should receive a uterotonic soon after delivery to prevent hemorrhage.^34^^,^^35^ Likewise, although immediate skin-to-skin contact was not common practice, with coaching support birth attendants recognized tangible improvements in babies' status, including better temperature regulation and easier initiation of breastfeeding. A third behavior with visible benefits was for the birth attendant to check the mother for bleeding after delivery and before discharge. Although postpartum hemorrhage is a well-known cause of maternal mortality, routinely checking for bleeding in the mother was frequently skipped. With coaching reminders, birth attendants saw the value of routinely assessing bleeding in order to recognize hemorrhage early.

Experiencing immediate, visible benefits from a new practice increases the likelihood that an individual will repeat the new practice.

### Slow or Resistant Change

Checklist-related behaviors that required extra effort with little visible benefit were more challenging for coaches to gain birth attendants' buy-in, even if there was a well-researched reason or mandate to comply. For example, lack of compliance with hand hygiene practices is a well-known issue for health care workers.^36^ We found that birth attendants would wash hands if a coach was physically present, but when a coach was not present birth attendants performed hand hygiene on only 36% of occasions before delivery. Although hand hygiene is critical for infection prevention,^37^ symptoms generally do not occur until after a mother and baby have been discharged from the facility and no longer are receiving care from the birth attendants. Thus, lack of a clear, immediate benefit from handwashing as well as the additional time and effort needed to perform the behavior likely limited the sustainability of improvements in handwashing.

Behaviors that required extra effort with little visible benefit were more challenging for coaches to gain birth attendants' buy-in.

Likewise, using a thermometer to measure a mother's or baby's temperature was not current practice at many facilities at the beginning of the intervention. Although adherence with this practice with the coach present was 95% for mother's temperature and 91% for baby's temperature after just 2 months of the intervention, when coaches were not present, temperature was measured for mothers in only 54% of occasions and in babies in only 42% of occasions. Field staff reported that birth attendants commonly used their hand to subjectively feel if a patient had a fever and were satisfied with this method, especially if a thermometer was missing. Additionally, many facilities experienced unreliable electricity, and thermometers are difficult to read in dark rooms. The increased complexity of using a thermometer instead of tactile approximation in addition to the difficulty of seeing the thermometer in a dark room represent 2 unfortunate barriers that have reduced adoption of new behaviors.^33^

### Importance of Leadership Support

The SCC is included as part of the Government of India Maternal and Newborn Health Toolkit^24^; however, it has not been widely used in Uttar Pradesh and was not in use at any participating facility at the start of the trial. Formally ensuring state- and district-level administrative support for the program and the subsequent 2-day training for facility staff to launch the BetterBirth Program at each facility may have contributed to the high initial adherence to many essential birth practices during the first month of the intervention (mean: 77%; range: 45% to 98%). This is especially relevant given the low level of birth attendants' adherence to essential practices found in similar studies in the region.^38^

### Systems-Level Incentives

Certain essential birth practices were practiced in most facilities from the beginning of the intervention due to governmental oversight and required documentation. For example, measuring a baby's weight following delivery is a standard requirement in birth registries and thus was frequently performed. Breastfeeding, although also part of a governmental promotion strategy, did not require specific documentation and thus did not have the same high level of adherence. However, behaviors such as breastfeeding practices were responsive to coaching and increased during the intervention, with coaches reporting that this occurred because they were able to reinforce the importance of the practice due to the clear governmental guidelines.

Conversely, incentives at the facility and district level to minimize waste may have contributed to less consistent administration of BCG and other vaccines. Each vial contained 10 doses, and birth attendants were observed to be avoiding administration unless a number of babies were present at once in order to not waste vaccine. Due to limitations in data collection, we did not know how many babies returned to the facility at a later date to receive the vaccines.

Some birth practices were practiced in most facilities from the beginning due to governmental oversight and required documentation, such as measuring baby's weight after delivery.

### Integrating the SCC Into Existing Workflows

Encouraging the habit of SCC use had mixed success. The more structured patient assessments that occurred on admission and within 1 hour after birth were conducive to SCC use. However, just before delivery (pause point 2) is an extremely busy time for birth attendants; birth attendants frequently regarded referring to a checklist as more of a burden or barrier to providing timely care at this point. Because the SCC was not always well integrated into the existing patient record (“bedhead ticket”), it was easily overlooked. Practices that improved at pause point 2, namely ensuring the necessary supplies were ready at the bedside, likely improved through advance preparation of birth trays with all necessary supplies at the start of the shift.

### Limitations

This analysis had a number of limitations. Given the high level of adherence reported by coaches, it is possible that coaches may have overstated birth attendants' adherence due to social desirability or fear of bad reviews if improvement was not seen. Additionally, although we assume that adherence to essential practices recorded by an independent observer reflects normal day-to-day care, it is possible that we experienced a Hawthorne-like effect in our measurements. However, Leonard and Masatu found that while having an observer present will cause clinicians to positively change their behavior when first observed, clinicians return to their typical practices after 10 to 15 observations.^39^ Since BetterBirth staff spent substantial time at each facility, this may limit the influence of any Hawthorne-like effect. To minimize bias, we could have installed video cameras or another passive observation tool,^40^ but this approach was not cost-effective for the scale of the intervention and likely would not have been accepted by facility staff, patients, or the Institutional Review Boards reviewing the trial's ethical procedures.

Observations were structured to record only whether clinical practices occurred, such as taking blood pressure and listening for fetal heart sounds. We did not determine the accuracy of measures taken by birth attendants or whether clinical practices, such as neonatal resuscitation, were conducted correctly. A forthcoming analysis will look at whether the intervention was associated with reductions in maternal and neonatal mortality and maternal morbidity.^20^ Additionally, for this analysis we did not focus on the ultimate receivers of care—the patients themselves—who may perceive the performance of essential practices and their overall quality of care differently.

Coaches and independent observers were not able to document adherence to essential practices at all pause points for all deliveries. For example, neither coaches nor independent observers made observations at night due to safety concerns for study staff; practices at night may be different than those during the day. Coaches worked with birth attendants and facility staff in all intervention facilities to standardize discharge procedures, to improve the quality of recovery room offerings, and to encourage women and their families to stay for the minimally recommended 24 hours post-birth. Nevertheless, many women left facilities within 6 hours after birth due to family pressures and lack of food availability, and these departures were generally against medical advice. Because women often leave without informing facility staff, it was not possible for independent observers to observe discharge procedures in a standard manner across all study facilities.

Due to the small number of facilities included in the subanalysis, we were unable to confidently assess if particular facility characteristics or programmatic factors were associated with these differences. In comparing the coach-recorded adherence rate in the 15 facilities of the subanalysis versus the 45 facilities not in the subanalysis, we found no major differences between the 2 groups.

### Future Research

While we advanced the understanding of how and when checklists are used in the presence or absence of a peer-coach, there are many areas to explore further. As part of the randomized controlled trial, the BetterBirth Program used a standard number of coaching visits with a prescribed frequency across all facilities. Further inquiry is needed on how the frequency and length of coaching can be structured to maximize sustained behavior change. Additional research is also needed to assess if there is a threshold level of adherence to essential practices that would be associated with improvement in health outcomes since consistent and complete adherence to the SCC and essential birth practices may not be likely in standard clinical practice. More research is needed to understand if there are specific programmatic, facility, or maternal characteristics that account for differences in adherence and how the SCC with coaching could operate as a team-based vs. individual-level intervention for health care workers. Sustainability will be assessed in a forthcoming analysis that explores adherence to essential practices 12 months after the start of the coaching program. Finally, a cost analysis of delivering this intervention in the context of Uttar Pradesh is currently underway.

Further research is needed to discover how the frequency and length of coaching can be structured to maximize sustained behavior change.

## CONCLUSION

We conclude that coaching was effective in increasing the uptake of birth attendants' essential birth practices when a coach was present, but adherence to some behaviors was reduced when the coach was absent. These findings will help to optimize the use of peer coaches and improve overall implementation of the SCC at future facilities, to improve the quality of care available during childbirth, and to understand how to improve behavior change interventions with health care workers.

## Supplementary Material

Supplement 1

## Notes

**Peer Reviewed**
