# Supplementary material for: Improving Adherence to Essential Birth Practices Using the WHO Safe Childbirth Checklist With Peer Coaching: Experience From 60 Public Health Facilities in Uttar Pradesh, India
Source: Glob Health Sci Pract. 2017 Jun 27;5(2):217–31. doi: 10.9745/GHSP-D-16-00410 (PMC5487085; doi:10.9745/GHSP-D-16-00410)
Supplement: Supplement 1 [file GHSP-D-16-00410_index.html]

Supplement to Improving Adherence to Essential Birth Practices Using the WHO Safe Childbirth Checklist With Peer Coaching: Experience From 60 Public Health Facilities in Uttar Pradesh, India | Global Health: Science and Practice

## Supplements

SUPPLEMENT 1. Characteristics of Intervention Facilities (N=60), Coaching Visits, and Childbirth Quality Coordinators (CQCs), Uttar Pradesh, India, December 2014 to September 2016

SUPPLEMENT 2. Adherence of Birth Attendants to 43 Essential Birth Practices Among 5,971 Deliveries, Documented by Coaches in 60 Intervention Facilities Across the 8-Month BetterBirth Intervention, Uttar Pradesh, India

SUPPLEMENT 3. Adherence of Birth Attendants to Essential Birth Practices, Documented by Independent Observers Versus Coaches in 15 Intervention Facilities After 2 Months of Receiving the BetterBirth Intervention, Uttar Pradesh, India

**Files in this Data Supplement:**

- Supplement 1
- Supplement 2
- Supplement 3
