# Supplementary material for: Improving Adherence to Essential Birth Practices Using the WHO Safe Childbirth Checklist With Peer Coaching: Experience From 60 Public Health Facilities in Uttar Pradesh, India
Source: Glob Health Sci Pract. 2017 Jun 27;5(2):217–31. doi: 10.9745/GHSP-D-16-00410 (PMC5487085; doi:10.9745/GHSP-D-16-00410)
Supplement: Supplement 1 [file 16-00410-Firestone-Supplement_1.pdf]

Marx Delaney M, Maji P, Kalita T, et al. Improving adherence to essential birth practices using the WHO Safe Childbirth Checklist with peer coaching: experience from 60 public health facilities in Uttar Pradesh, India. *Glob Health Sci Pract*. 2017;5(2). https://doi.org/10.9745/GHSP-D-16-00410

**SUPPLEMENT 1.** Characteristics of Intervention Facilities (N=60), Coaching Visits, and Childbirth Quality Coordinators (CQCs), Uttar Pradesh, India, December 2014 to September 2016

| | |
|---|---|
| **Facility Characteristics** | |
| Total number of facilities | 60 |
| Annual number of deliveries, mean (95% CI) | 1,599 (1,486, 1,712) |
| Number of birth attendants per facility, mean (95% CI) | 4.4 (4.1, 4.7) |
| Distance to district hospital, km, mean (95% CI) | 29.5 (26.0, 33.1) |
| Facility type, % | |
| Primary health center | 38% |
| Community health center | 45% |
| First referral unit | 17% |
| **Coaching Visits** | |
| Expected number of coach visits | 43 |
| Completed number of coach visits, mean (%) | 42.1 (97.9%) |
| Expected number of team leader visits | 23 |
| Completed number of team leader visits, mean (%) | 21.6 (93.9%) |
| **CQCs** | |
| Total number of facility staff trained as CQCs across 60 facilities | 109 |
| Number of facilities with 1-, 2-, and 3- person CQC teams, n (%) | |
| 1-person CQC | 15 facilities (25%) |
| 2-person CQC team | 41 facilities (68%) |
| 3-person CQC team | 4 facilities (7%) |
| Type of staff appointed to CQC role, n (%) | |
| Auxiliary nurse-midwife | 5 (4.6%) |
| Staff nurse | 25 (22.9%) |
| Lady Medical Officer (Doctor) | 34 (31.2%) |
| Medical Officer (Doctor) | 23 (21.1%) |
| Pharmacist | 12 (11.0%) |
| Other delivery support staff | 2 (1.8%) |
| Other administrative staff | 8 (7.3%) |

Abbreviations: CI, confidence interval, CQC, childbirth quality coordinator.
