# Supplementary material for: Improving Adherence to Essential Birth Practices Using the WHO Safe Childbirth Checklist With Peer Coaching: Experience From 60 Public Health Facilities in Uttar Pradesh, India
Source: Glob Health Sci Pract. 2017 Jun 27;5(2):217–31. doi: 10.9745/GHSP-D-16-00410 (PMC5487085; doi:10.9745/GHSP-D-16-00410)
Supplement: Supplement 1 [file 16-00410-Firestone-Supplement_2.pdf]

Marx Delaney M, Maji P, Kalita T, et al. Improving adherence to essential birth practices using the WHO Safe Childbirth Checklist with peer coaching: experience from 60 public health facilities in Uttar Pradesh, India. *Glob Health Sci Pract*. 2017;5(2). https://doi.org/10.9745/GHSP-D-16-00410

SUPPLEMENT 2. Adherence of Birth Attendants to 43 Essential Birth Practices Among 5,971 Deliveries, Documented by Coaches in 60 Intervention Facilities Across the 8-Month BetterBirth Intervention, Uttar Pradesh, India

Level of Improvement	Behavior	Overall			Month 1 (Weeks 1 to 4)	Months 7&8 (Weeks 25 to 32)[a]	Absolute Difference (Months 7&8–Month 1)	OR	95% CI		*P* Value
		n	N	%							
Minimal improvement (<15 percentage-point change, Months 7&8–Month 1)	Gloves worn for vaginal exam	2212	2233	99%	98%	100%	2 points	1.56	1.05	2.31	.03
	Glove use at birth	2304	2350	98%	96%	99%	3 point	1.28	0.94	1.75	.11
	Vaginal exam done on admission	2227	2258	99%	97%	100%	3 points	1.37	1.08	1.73	.01
	Baby's breathing evaluated after birth	2316	2350	99%	96%	99%	3 points	1.51	1.09	2.10	.01
	Oral polio vaccine given	1814	2091	87%	86%	89%	3 points	1.02	0.91	1.16	<.001
	Cord ligature available at bedside	2287	2334	98%	94%	99%	5 points	1.66	1.01	2.75	.047
	Gloves available at bedside	2280	2334	98%	93%	99%	6 points	1.66	1.08	2.55	.02
	Blade available at bedside	2273	2334	97%	91%	99%	8 points	1.83	1.04	3.20	.04
	Mucus extractor available at bedside	2256	2334	97%	89%	98%	9 points	1.63	1.08	2.47	.02
	Baby's weight	2190	2359	93%	85%	95%	10 points	1.34	1.12	1.62	.001
	Oxytocin available at bedside	2196	2334	94%	87%	98%	11 points	1.49	1.22	1.82	<.001
	Pads available at bedside	2215	2334	95%	86%	98%	12 points	1.66	1.22	2.26	.001
	Mother's blood pressure taken on admission	2108	2258	93%	85%	97%	12 points	1.37	1.00	1.89	.05
	Bacille Calmette-Guérin vaccine given	1605	2091	77%	75%	87%	12 points	1.09	0.99	1.21	.07
Moderate improvement (15–24 percentage-point change,	Mother's temperature taken before discharge	1907	2091	91%	80%	95%	15 points	1.53	1.30	1.81	<.001
	Clean towel available at bedside	2139	2334	92%	81%	98%	17 points	1.47	1.20	1.80	<.001
	Check baby feeding before discharge	1873	2091	90%	81%	98%	17 points	1.38	1.22	1.55	<.001

Marx Delaney M, Maji P, Kalita T, et al. Improving adherence to essential birth practices using the WHO Safe Childbirth Checklist with peer coaching: experience from 60 public health facilities in Uttar Pradesh, India. *Glob Health Sci Pract*. 2017;5(2). https://doi.org/10.9745/GHSP-D-16-00410

Level of Improvement Months 7&8–Month 1)	Behavior	Overall			Month 1 (Weeks 1 to 4)	Months 7&8 (Weeks 25 to 32)[a]	Absolute Difference (Months 7&8–Month 1)	OR	95% CI		*P* Value
		n	N	%							
	Baby's temperature taken before discharge	1845	2091	88%	76%	94%	18 points	1.41	1.23	1.61	<.001
	Hand hygiene before delivery	2076	2309	90%	76%	94%	18 points	1.46	1.18	1.81	<.001
	Oxytocin given 1 minute after birth	2167	2350	92%	81%	99%	18 points	1.66	1.38	2.00	<.001
	Check bleeding before discharge	1839	2091	88%	77%	96%	19 points	1.25	1.08	1.45	.002
	Neonatal bag and mask available at bedside	2133	2334	91%	78%	99%	21points	1.80	1.35	2.40	<.001
	Family planning discussed	1886	2091	90%	77%	98%	21 points	1.58	1.34	1.87	<.001
	Mother's blood pressure taken after delivery	2067	2359	88%	71%	93%	23 points	1.52	1.25	1.84	<.001
	Skin-to-skin care immediately after birth	2041	2350	87%	71%	94%	23 points	1.43	1.26	1.62	<.001
	Check baby breathing before discharge	1843	2091	88%	74%	97%	23 points	1.57	1.39	1.79	<.001
Major improvement (≥25 percentage-point change, Months 7&8–Month 1)	Check bleeding after delivery	2094	2359	89%	72%	97%	25points	1.64	1.33	2.02	<.001
	Breastfeeding initiation	2056	2359	87%	68%	95%	27 points	1.52	1.33	1.74	<.001
	Mother's blood pressure taken before delivery	1620	2334	69%	53%	80%	27 points	1.23	1.10	1.38	<.001
	Danger signs explained to mother or birth companion before discharge	1908	2091	91%	71%	98%	27 points	2.04	1.70	2.44	<.001
	Hand hygiene before vaginal exam	1935	2233	87%	71%	99%	28 points	1.82	1.41	2.35	<.001
	Mother's temperature on admission	1992	2258	88%	71%	99%	28 points	1.76	1.39	2.22	<.001
	Skin-to-skin at 1 hour	1964	2359	83%	64%	93%	29 points	1.52	1.32	1.75	<.001
	Mother's temperature taken after delivery	2024	2359	86%	65%	95%	30 points	1.73	1.46	2.06	<.001
	Fetal heart sound measurement	1805	2258	80%	62%	97%	35 points	1.53	1.29	1.82	<.001

Marx Delaney M, Maji P, Kalita T, et al. Improving adherence to essential birth practices using the WHO Safe Childbirth Checklist with peer coaching: experience from 60 public health facilities in Uttar Pradesh, India. *Glob Health Sci Pract*. 2017;5(2). https://doi.org/10.9745/GHSP-D-16-00410

Level of Improvement	Behavior	Overall			Month 1 (Weeks 1 to 4)	Months 7&8 (Weeks 25 to 32)[a]	Absolute Difference (Months 7&8–Month 1)	OR	95% CI		*P* Value
		n	N	%							
	Mother's temperature taken before delivery	1556	2334	67%	46%	81%	35 points	1.30	1.17	1.44	<.001
	Baby's temperature taken after delivery	1921	2359	81%	57%	93%	36 points	1.66	1.44	1.91	<.001
	Danger signs explained to mother or birth companion after delivery	1716	2359	73%	54%	92%	38 points	1.67	1.38	2.04	<.001
	Danger signs explained to mother or birth companion on admission	1525	2258	68%	45%	96%	51 points	1.77	1.48	2.14	<.001
**Checklist use**	Checklist use at admission	2133	2258	94%	84%	98%	14 points	1.72	1.23	2.42	.002
	Checklist use before delivery	2038	2334	87%	66%	94%	28 points	1.58	1.24	2.02	<.001
	Checklist use after delivery	2173	2359	92%	75%	95%	20 points	1.69	1.27	2.27	<.001
	Checklist use before discharge	2028	2091	97%	90%	99%	9 points	1.80	1.37	2.36	<.001

Abbreviations: CI, confidence interval; OR, odds ratio.

[a] Data for months 7 and 8 were combined due to infrequency of observation.
