# Supplementary material for: Improving Adherence to Essential Birth Practices Using the WHO Safe Childbirth Checklist With Peer Coaching: Experience From 60 Public Health Facilities in Uttar Pradesh, India
Source: Glob Health Sci Pract. 2017 Jun 27;5(2):217–31. doi: 10.9745/GHSP-D-16-00410 (PMC5487085; doi:10.9745/GHSP-D-16-00410)
Supplement: Supplement 1 [file 16-00410-Firestone-Supplement_3.pdf]

Marx Delaney M, Maji P, Kalita T, et al. Improving adherence to essential birth practices using the WHO Safe Childbirth Checklist with peer coaching: experience from 60 public health facilities in Uttar Pradesh, India. *Glob Health Sci Pract*. 2017;5(2). https://doi.org/10.9745/GHSP-D-16-00410

SUPPLEMENT 3. Adherence of Birth Attendants to Essential Birth Practices, Documented by Independent Observers Versus Coaches in 15 Intervention Facilities After 2 Months of Receiving the BetterBirth Intervention, Uttar Pradesh, India

Absolute Difference in Adherence Recorded by Coach and Independent Observer	Behavior	Coach			Independent Observer			Absolute Difference (% Coach–% Independent Observer)
		Completed	Observed	%	Completed	Observed	%	Percentage points
Minimal difference (<15 percentage points)	Cord ligature available at bedside	313	316	99%	1023	1027	100%	-1 point
	Neonatal bag and mask available at bedside	308	316	97%	979	1027	95%	2 points
	Pads available at bedside	302	316	96%	954	1027	93%	3 points
	Mucus extractor available at bedside	312	316	99%	978	1027	95%	4 points
	Baby weighed within 1 hour	335	348	96%	896	996	90%	6 points
	Clean towel available at bedside	287	316	91%	863	1027	84%	7 points
	Glove use at birth	327	332	98%	924	1027	90%	8 points
	Skin-to-skin immediately after birth	306	332	92%	787	996	79%	13 points
Moderate difference (15 to 24 percentage points)	Oxytocin given 1 minute after birth	319	332	96%	813	1026	79%	17 points
	Sterile scissors/blade available at bedside	312	316	99%	830	1027	81%	18 points
	Breastfeeding within 1 hour	323	348	93%	693	996	70%	23 points
Major difference (≥25 percentage points)	Mother's blood pressure taken on admission	259	273	95%	600	1041	58%	37 points
	Mother's temperature taken on admission	258	273	95%	564	1041	54%	41 points
	Baby's temperature taken within 1 hour	316	348	91%	422	996	42%	49 points
	Hand hygiene	292	316	92%	374	1027	36%	56 points
Checklist use	Checklist use after birth	332	348	95%	739	996	74%	21 points
	Checklist use on admission	261	273	96%	595	1041	57%	39 points
	Checklist use before delivery	272	316	86%	252	1027	25%	61 points
	**Grand Total**	**5727**	**6051**	**95%**	**13724**	**19399**	**71%**	**24 points**
